# Improved Depth-of-Field Photoacoustic Microscopy with a Multifocal Point Transducer for Biomedical Imaging

**DOI:** 10.3390/s20072020

**Published:** 2020-04-03

**Authors:** Thanh Phuoc Nguyen, Van Tu Nguyen, Sudip Mondal, Van Hiep Pham, Dinh Dat Vu, Byung-Gak Kim, Junghwan Oh

**Affiliations:** 1 Department of Mechatronics, Cao Thang Technical College, Ho Chi Minh City 700000, Vietnam; 2 Interdisciplinary Program of Biomedical Mechanical and Electrical Engineering, Pukyong National University, Busan 48513, Korea; nguyen.vantu91@gmail.com (V.T.N.); pvhiep.mta.hut@gmail.com ( V.H.P.); dinhdatvn96@gmail.com (D.D.V.); 3 Center for Marine-Integrated Biomedical Technology, Pukyong National University, Busan 48513, Korea; mailsudipmondal@gmail.com; 4 College of Future Convergence, Pukyong National University, Busan 48513, Korea; bgkim@pknu.ac.kr

**Keywords:** ultrasound, photoacoustic imaging, photoacoustic microscopy, biomedical imaging, multifocal point transducer

## Abstract

In this study, a photoacoustic microscopy (PAM) system based on a multifocal point (MFP) transducer was fabricated to produce a large depth-of-field tissue image. The customized MFP transducer has seven focal points, distributed along with the transducer’s axis, fabricated by separate spherically-focused surfaces. These surfaces generate distinct focal zones that are overlapped to extend the depth-of-field. This design allows extending the focal zone of 10 mm for the 11 MHz MFP transducer, which is a great improvement over the 0.48 mm focal zone of the 11 MHz single focal point (SFP) transducer. The PAM image penetration depths of a chicken-hemoglobin phantom using SFP and MFP transducers were measured as 5 mm and 8 mm, respectively. The significant increase in the PAM image-based penetration depth of the chicken-hemoglobin phantom was a result of using the customized MFP transducer.

## 1. Introduction

Of late, optical techniques have been used widely in biomedical imaging, which has improved the performance of in vivo diagnosis with high optical contrast [1,2]. This approach produces strong light scattering effects and a low spatial resolution. The optical microscopy penetration depth is limited to approximately 1 mm. Photoacoustic (PA) imaging (PAI) is a biomedical imaging modality based on the PA effect [3,4,5,6,7]. When short-pulsed laser light is directed on tissues, chromophores absorb some of the light energy that is converted into acoustic waves due to rapid thermal expansion. Photoacoustic microscopy (PAM) is also widely used to image tissues through optical absorption [8,9,10,11,12,13]. PAM can image optical contrast beyond the existing depth limit for high-resolution optical imaging [14]. The spatial resolution depends on the performance of the ultrasonic transducer. Many studies developed photoacoustic tomography by employing a short pulse to generate ultrasound waves in biological tissues; such techniques are used for in vivo biomedical imaging [1,15].

Ultrasonic transducers play an important role in PAI systems [1,13,16,17]. The transducer’s parameters have a significant effect on image quality [18]. The main parameters of ultrasonic transducers are center frequency, bandwidth, focal length, focal zone, aperture size, and lateral and axial resolutions. Single focal point (SFP) transducers have a limited focal zone so as to acquire a deep image. Ultrasonic array transducers can control focal depths through dynamic focusing algorithms to capture the target image [19,20,21,22]. However, the fabrication process of this type of transducer is highly complicated. Multifocal point (MFP) transducers, which are developed to be used in imaging systems, are proposed to increase the image depth in ultrasound imaging [23]. The newly designed MFP transducers show a significant increase in focal zone compared with SFP transducers. Combined with PA systems, the penetration depth in deep imaging can be increased. 

In PAI systems, the depth of penetration depends on the frequency of the ultrasonic wave. Higher frequencies have a small depth of penetration, whereas lower frequencies have a greater depth of penetration. To obtain the correct depth of an image, PAM requires an appropriate high frequency with a short wavelength and an enhanced resolution. Previous studies reported the use of phased array transducers for the PAI systems with complex dynamic focusing algorithms to acquire images [5,21,24,25,26,27,28,29]. Chulhong et al. [5] imaged biological tissues using a combined handheld PA microimaging probe and a transducer array. The lymph nodes containing methylene blue at a depth of 4.5 cm in tissue were visible in the PA images. Vogt et al. [21] used four clinical ultrasound transducer arrays in a PAI system with frequencies of 2.5, 8, 8.7, and 12.4 MHz. The largest penetration depths in the PAI system were obtained from the transducer with the lowest frequency, i.e., 2.5 MHz. A real-time 512-channel PA system with a 5 MHz transducer array imaged mouse brain vasculature at a depth of 4.5 mm with a lateral resolution of 200 µm [25]. However, a combination of two systems poses complications for setup and operation.

In addition, for array ultrasound signals, the complex algorithm of the synthetic-aperture focusing technique (SAFT) had been applied to acquire qualified final images [21,25,26,27,29]. This study was conducted with the motive to design a custom-made MFP transducer with seven focal points and a long focal zone for deeper imaging applications, which does not require the application of SAFT for reconstruction or any post-processing to obtain the image. Because MFP transducers are designed using a single piezoelectric element, it is easy to acquire the image using only a single-channel ultrasonic pulser/receiver. 

The contributions of this study are presented in three aspects. First, two types of focused transducer (SFP and MFP) were designed and fabricated, both made of the same type of 28 µm polyvinylidene fluoride (PVDF) film and producing the same center frequency. In the case of the SFP transducer, with only one focal point, a large image depth cannot be obtained owing to the limited size of the focal zone. The main objective of this study was to expand the MFP transducer’s focal zone, which may create many focal points at different depths with the use of a multi-spherical pattern (MSP) model. Second, the proposed design of the MFP transducer can be driven by a one-channel ultrasonic pulser/receiver system because of its single element function. This enables simple operation and data processing to acquire images without applying the complex SAFT as the transducer array system. Third, the significant difference between the focal zone and penetration depth of images from two transducers was distinguished in ultrasound imaging of the wire phantom, PAI of the needle, and hemoglobin (Hb) embedded in chicken tissues.

## 2. Materials and Methods

### 2.1. Transducer Design Materials

The piezoelectric element is the most important element of an ultrasonic transducer, which is made of piezoelectric polymers. PVDF membrane (Piezotech S.A.S, France) is the preferred polymer and has been extensively studied for many decades in the manufacture of high-frequency transducers [23,30,31,32,33]. PVDF is a special material used in high-purity applications, as well as in solvents, acids, and hydrocarbon resistance. Polymerization, stretching, and polling processes for a 28 µm PVDF element can be applied for developing transducers. In this study, PVDF was selected to fabricate SFP and MFP transducers due to its advanced properties. Table 1 details the properties of PVDF used in this study. Although PVDF film’s acoustic impedance (~4 MRayl) is lower than that of piezoceramics and crystal materials, PVDF shows a good mechanical versatility, which makes it easy to press the film into a spherical shape. The transducer developed from this PVDF has a normal broad bandwidth. In addition, PVDF film has a small dielectric constant appropriate for electrical impedance matching [34].

### 2.2. Transducer Design

**Figure 1:**
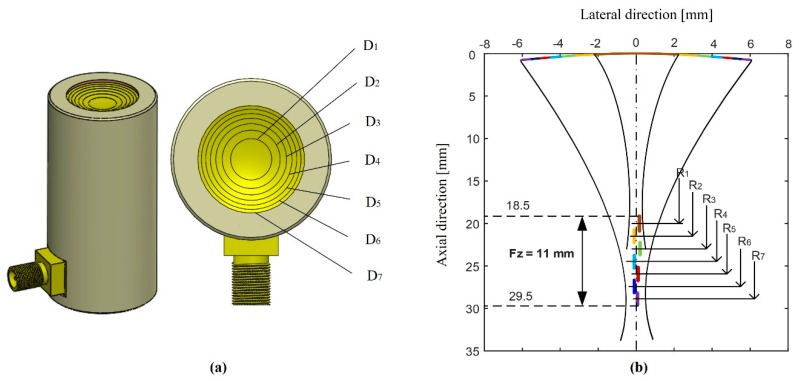
The (**a**) profile and (**b**) distribution of the focal zones of the multifocal point (MFP) transducer.

SFP transducers have a functional limitation in the focal zone and penetration depth. To extend the length of the focal zone, the multifocal point transducer was designed with a focal zone of 11 mm. In this study, the structure of the MFP transducer was designed similarly to the design reported in a previous study [23]. Figure 1 shows the profile and focal zones distribution of the developed transducer. The surface of the MFP transducer was designed by connecting seven parts with same areas in order to create the same level of intensity in their focused areas. The “*Ri*” is the radius of part “*i*” (*i* = 1–7). The distance between two focal points *b* = *R_j_* –*R_j-1_* = 1.5 mm (*j* = 2–7) The parameters of the seven-focal-point transducer were designed as shown in Table 2. 

The most important feature in Table 2 shows that a longer focal length obtains a smaller F-number. To obtain the best axial resolution, the system needs the smallest F-number for the best quality image (Axial resolution = speed of sound x F-number/center frequency). The seventh part of the MFP transducer has the smallest F-number of 2.3 and the longest focal length of 28.87 mm, which can obtain better axial resolutions at deeper depths.

**Figure 2:**
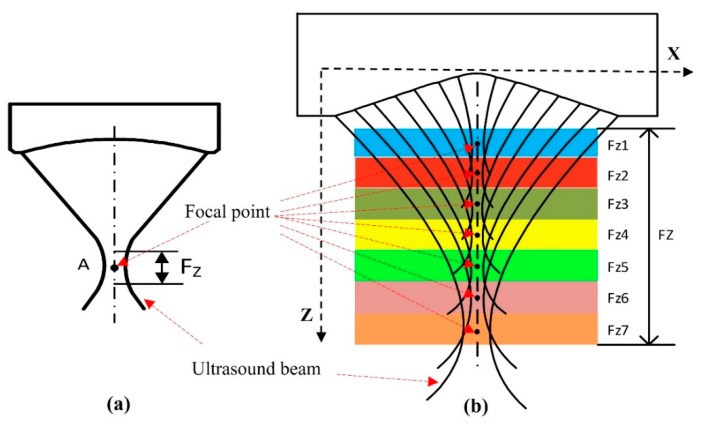
The distribution of the focal points and focal zones of (**a**) the single focal point (SFP) and (**b**) MFP transducer.

Figure 2 shows the comparison of the transducers’ focal zones. For the SFP transducer, the front face was formed by a steel ball bearing of 12.7 mm in radius. The SFP transducer has only one focal point at the focus depth of 12.7 mm and only one focal zone of 0.48 mm. The front surface’s parameter of the MFP transducer was designed and simulated using the Matlab (version 2013a, Mathworks, Natick, MA) software. The MFP transducer has seven focal points, which created seven focal zones.

The parameters of the MFP transducer were carefully calculated to obtain tightly focused areas from the first to the last focal zone. Table 3 shows the overlap length between the two closed focal zones, which confirms that the focal depths are continued on the completely focused areas. Based on the distribution of the focal zones, the MFP transducer can capture a good target image as the object is placed in the area of the focal zone between 18.50 mm and 29.50 mm.

### 2.3. Transducer Fabrication

**Figure 3:**
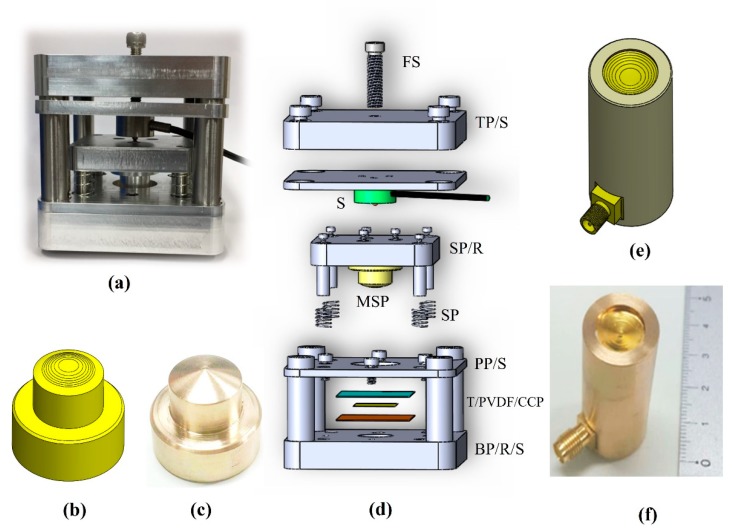
(**a**) A photograph of the press-fit structure; (**b**) the designed multi-spherical pattern (MSP); (**c**) a photograph of the MSP; (**d**) the press-fit’s components: base plate/rod/screw (BP/R/S), Teflon/PVDF/copper-clad polyimide (T/PVDF/CCP), pressure plate/screw (PP/S), spring (SP), MSP, slide plate/rod (SP/R), sensor of force (S), top plate/screw (TP/S), and force screw (FS); (**e**) the profile of MFP transducer; and (**f**) a photograph of the MFP transducer.

To acquire a large penetration depth image, a combination of the light absorption and acoustic detector was used. Two types of ring-shaped transducer (SFP and MFP) were developed to compare the different performances in PAM imaging. Figure 3 shows the device used for forming the multi-spherical surfaces of the MFP transducer. The press-fit system (Figure 3a) was fabricated with five aluminum plates and four stainless steel rods with screws. The parameters of the MSP (Figure 3b) with seven spherically-focused surfaces were simulated using Matlab, and the original computer numerical control (CNC) machine fabricated the transducer, as shown in Figure 3c. The components of the press-fit system were designed using Matlab and made by using the CNC machine, as shown in Figure 3d. The MFP transducer was designed using Solidworks, as shown in Figure 3e. Figure 3f shows a photograph of the fabricated MFP transducer with seven focal points. 

**Figure 4:**
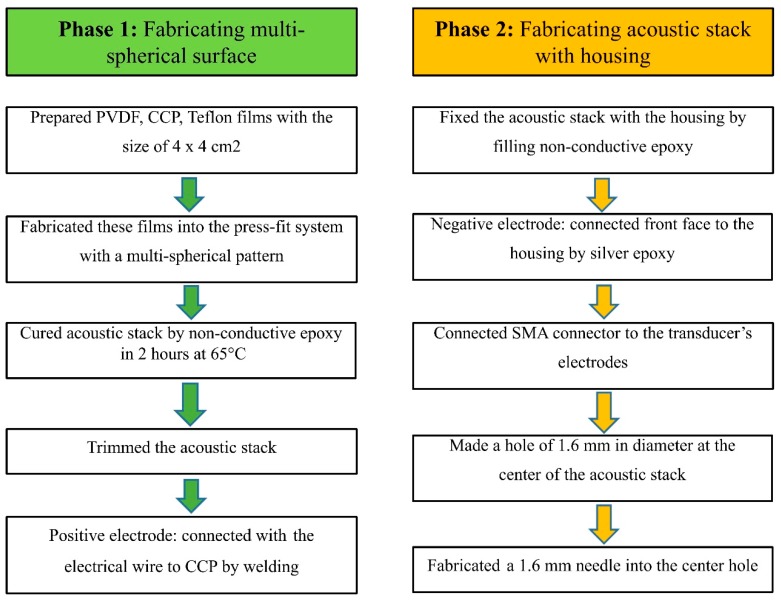
The fabrication process of the ring-shaped MFP transducer.

For the MFP transducer, the fabrication process proceeded in two phases, as shown in Figure 4. The press-fit system was used in the first phase to form a multi-spherical profile of the active membrane. The copper-clad polyimide (CCP; Hanwha Corp., FCCL, Korea), 28 µm PVDF membrane (Piezotech S.A.S, France), and Teflon films (4 × 4 cm) were prepared. For bonding the two films of CCP and PVDF, the epoxy (EPO TEK 301, Epoxy Technology, Billerica, MA, USA) was applied. To avoid tearing the films, springs were placed to minimize the vibration in the manufacturing process. The force sensor was controlled to display the tension value of the active film surface. The top and bottom plates were fixed using screws through rods. To ensure a uniform pressure on the surface of the MSP transducer, the forcing screw was rotated by a hexagonal bar wrench.

The press-fit system was inverted after these films were inserted into the base plate’s center hole. The Teflon tube was filled with the nonconductive epoxy to keep the spherical profile of the PVDF film after curing. The fabricated system was heated at 65 °C for 2 hours. After disassembling the press-fit system, the acoustic stack was taken out with an epoxy plug connected to it. The CCP and PVDF were trimmed close to the epoxy plug. The pin of the SMA connector (Mouser Electronics, TX, USA) was soldered to a small CCP line through an electrical wire. 

In the second phase, the acoustic stack was fabricated to a transducer housing. The acoustic stack was concentrically attached to the transducer housing. An open space was filled with a nonconductive epoxy inside the housing to keep the transducer’s long-term electrical and mechanical stability. Following the epoxy curing, the transducer housing was connected to the connector. A piece of silver epoxy (H20 epoxy, Epoxy Technology, Inc., USA) was cast between a piece of PVDF and the housing to create a ground path. A drill was used to form a hole of 1.6 mm at the center of the transducer’s surface, and a 14G needle (Syringe needle, Anhui, China) was then inserted into the hole with a thin layer of UV adhesive (Norland products, Inc, Cranbury, NJ, USA) to maintain the spherical form of the PVDF film. The laser cable was inserted inside the needle and adjusted at the transducer’s focal point to obtain the best resolution PA image. 

**Figure 5:**
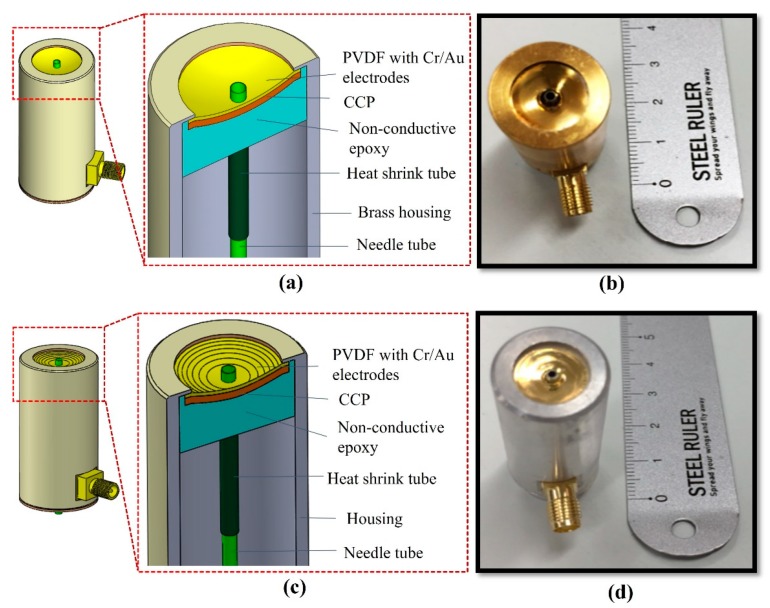
(**a**) A cross sectional view and (**b**) SFP ring-shaped transducer photograph. (**c**) A cross sectional view and (**d**) MFP ring-shaped transducer photograph.

For the SFP transducer, a steel ball bearing (Hecto, Jiangsu, China) of 25.4 mm in diameter was used to form a single spherical surface, producing a focal point at 12.7 mm and one focal zone of 0.48 mm. The same 28 µm PVDF film was used to fabricate an SFP transducer with an aperture diameter of 12 mm. Figure 5 shows the transducers’ cross-sectional view and photographs of the ring-shaped transducers.

### 2.4. Phantom Fabrication

#### 2.4.1. Wire Phantom

**Figure 6:**
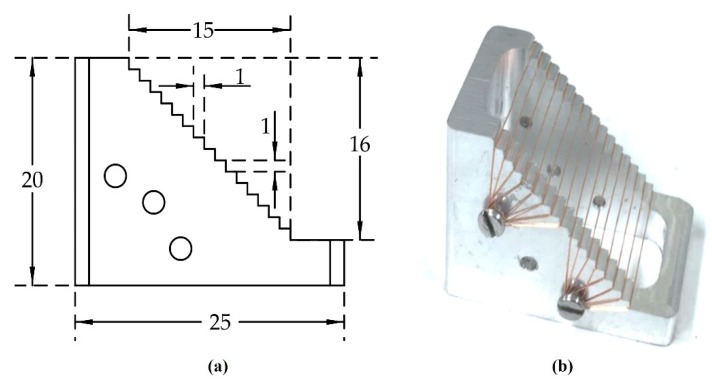
(**a**) The structure of the wire phantom. (**b**) A photograph of the wire phantom.

Sixteen phantom wires (25-µm) were placed diagonally, with an equal distance of 1 mm in the vertical axis and horizontal axis (Figure 6a). The transducer was moved along the X-axis to scan the wires image, which placed at the transducer’s focused position in degassed water. The reflected pulse-echo signal from the wire was used to figure the beam shape in the lateral direction. The final image was obtained by image processing, importing data into Matlab-based (Version. 2013a, Mathworks, Natick, MA, USA) software. 

#### 2.4.2. Chicken-Needle Phantom

**Figure 7:**
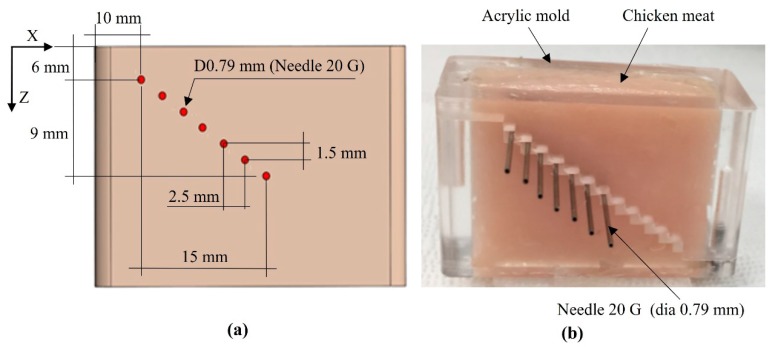
(**a**) The structure and (**b**) a photograph of the chicken-needle (CN) phantom.

Figure 7 shows the structure (Figure 7a) and photograph of a chicken-needle (CN) phantom. The chicken meat was placed into an acrylic mold, and 20G (syringe needle, Anhui, China) stainless steel needles (outer diameter, 0.79 mm) were inserted at different depths of the acrylic mold (Figure 7b). The needle phantom was composed of seven needles, which were separated by 1.5 mm in the Z-axis and 2.5 mm in the X-axis. The top surface of the chicken meat sample was flattened in the same plane as that of the top plane of the acrylic mold.

#### 2.4.3. Chicken-Hemoglobin Phantom

**Figure 8:**
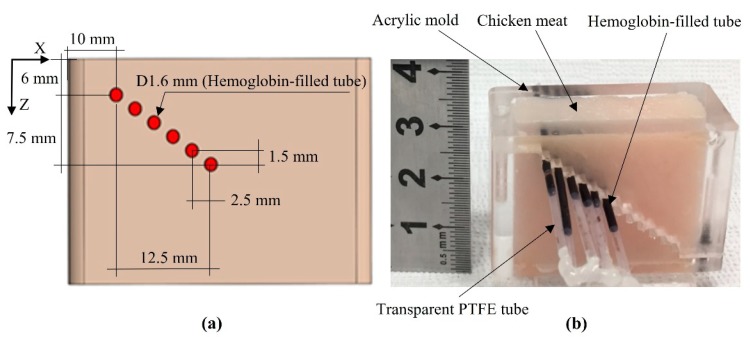
(**a**) The structure and (**b**) a photograph of the chicken-hemoglobin (CHb) phantom. The polytetrafluoroethylene (PTFE) tube with an inner diameter of 1.6 mm, wall thickness of 0.038 mm, and transparent color.

Figure 8 shows the structure (Figure 8a) and photograph of a chicken-hemoglobin (CHb) phantom, in which transparent polytetrafluoroethylene (PTFE) tubes (Zeus, Orangeburg, USA) containing Hb (Sigma-Aldrich, Merck, Seoul, South Korea) were embedded. The hemoglobin concentration of 13.6 g/dL is suitable for optical absorption coefficient of 4.0 cm^−1^ at 800 nm. The blood hemoglobin (Hb) concentration test is one of the most commonly performed tests; the normal Hb level in the human body ranges from 12 to 16 g/dL [21]. Hb is the iron-containing metalloprotein involved in the transport of oxygen that is found in nearly all vertebrates’ red blood cells, as well as in some invertebrates’ tissue. Hb in the blood transports oxygen to the whole body from the lungs or gills. Hb samples were injected into transparent tubes with a 1.6 mm outer diameter and 25 mm length. Hb-filled tubes were embedded at different depths of the chicken tissue in the acrylic mold (Figure 8b). To produce imaging targets at different depths, the CHb phantom comprised six Hb-filled tubes, which were positioned diagonally by 1.5 mm in the Z-axis and 2.5 mm in the X-axis.

### 2.5. Experimental Setup

#### 2.5.1. Ultrasound Imaging System

**Figure 9:**
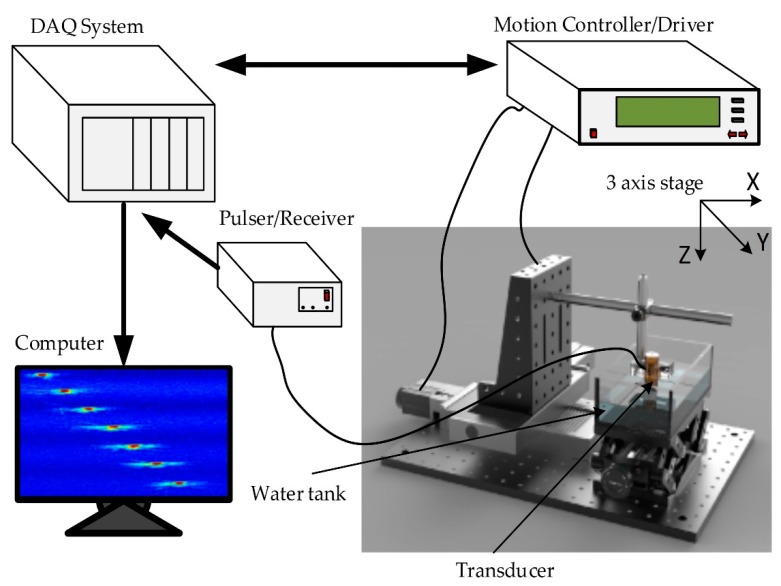
A schematic of the experimental ultrasound system.

Figure 9 shows a schematic diagram of the experimental process. A computer-controlled remote (DPR 500, JSR Ultrasonics, Pittsford, NY, USA) pulser/receiver and the transducer was connected to excite an electrical impulse at a 200 Hz repetition rate at 50 Ω damping with 3 µJ energy per pulse. To measure the pulse-echo and frequency spectra of the transducer, a glass plate was positioned at the focused position as a target. The reflected signal was received using a 500 MHz bandwidth receiver with a high pass filter of 5 MHz and a low pass filter of 500 MHz. The attained raw data were digitized at a sampling frequency of 500 megasamples/s. An 8-bit digitizer (NI PCI-5153EX, National Instruments, Austin, TX, USA) was used to digitize echoes. 

A stepper motor (UE63PP, Newport Corporation, CA, USA) was used to control the movement of the transducer, and a universal motion controller/driver (ESP300, Newport Corporation, CA, USA) was used to drive the motors’ motion. A LabView (LabView 2014, National Instrument, Austin, TX, USA) program was built to control all the processes mentioned above. A computer-controlled scanning stage was moved along the X-axis to acquire a B-scan image. 

An Agilent Keysight 4396B impedance analyzer (Agilent Technologies, Santa Clara, CA, USA) was used to measure the electrical impedance (magnitude and phase) of the fabricated SFP and MFP transducers. 

#### 2.5.2. Photoacoustic Microscopy System

**Figure 10:**
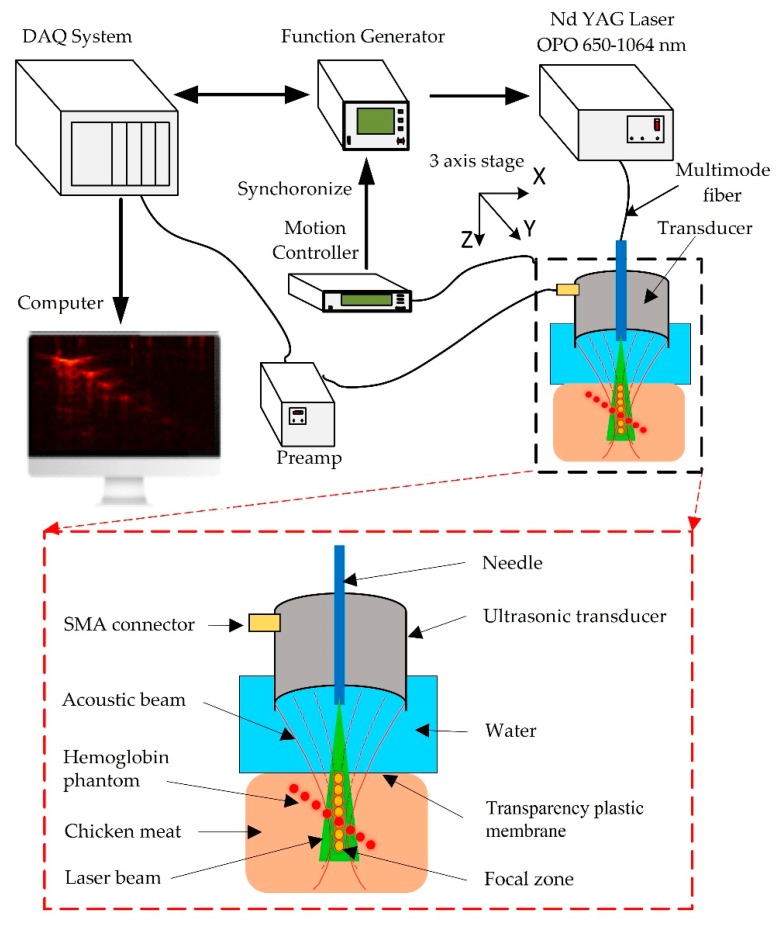
A schematic of the photoacoustic microscopy (PAM) experimental system. Laser OPO wavelengths: 650–1064 nm. Multimode fiber diameter: 1 mm, NA = 0.5.

The ex vivo experiments were conducted on the tissue phantom models. Figure 10 shows a schematic diagram of the experimental setup for the PAM system. Briefly, a tunable OPO laser (Surelite OPO Plus, Continuum, CA, USA) pumped by an Nd:YAG laser (Surelite III, San Jose, CA, USA) was applied as a light source with a 6 ns pulse width, 10 Hz repetition rate, and 650–1064 nm wavelength. A multimode fiber with a diameter of 1 mm and a light divergence of 30 degrees (NA = 0.5) was used to deliver the 800 nm pulsed laser beams with a laser energy of 0.18–1.98 mJ/pulse, which is well below the safety limit (20 mJ/cm^2^) of the American National Standards Institute. An 800 nm wavelength was employed to acquire the PA images. The input optical fiber was connected to a plano-convex (focal length: 50 mm; Thorlabs, Newton, NJ, USA). The fiber’s output end was connected to the custom transducers and aligned to the center of the illuminated area. To obtain the largest penetration depth in the PAM image, the fiber’s output end was adjusted to ensure a guaranteed maximum overlap area between the laser beam and the focal zone. The signals were then digitized and stored in coordination with a laser system, using a data acquisition (DAQ) system to capture the PA signals. The LabView program (Version 2012, National Instruments, Austin, TX, USA) was used to control the scanning procedure. Finally, through Hilbert’s transformation, the detected PA signals were transformed into PA images.

To evaluate the capability of transducers in PAM imaging, ex vivo experiments were conducted with both SFP and MFP transducers. Two types of tissue phantom were fabricated for the scanning system to obtain the PAM images, which demonstrated the differential penetration depths from two types of custom transducer. A tissue phantom was positioned inside a water tank through a thin transparent plastic membrane. An ultrasound gel was cast on the top plane of the tissue under the plastic membrane for acoustic coupling. The transducer was immersed in a tank of water during the experimental procedure. The B-scan mode was performed by linearly scanning the specimen along the transverse direction, which demonstrated the in-depth structure of the target. The large focal zone of the MFP transducer combined with the laser energy which can capture a deeper depth image with a high resolution. This is due to the seventh part of the MFP transducer which has the smallest F-number and the longest focal length related to the axial resolution.

## 3. Performance Evaluation 

### 3.1. Ultrasound Characterization

**Figure 11:**
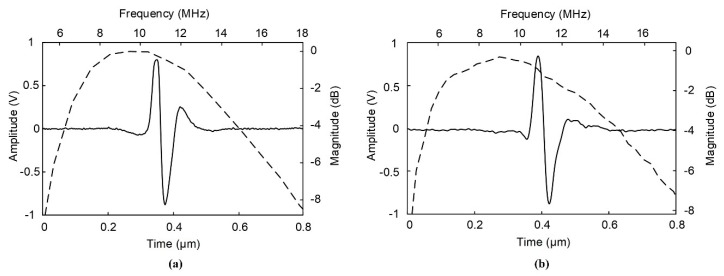
The measured pulse-echo and frequency spectra of transducers after forming a hole of (**a**) the single focal point transducer and (**b**) the seven-focal point transducer.

Figure 11 shows the measured pulse-echo response and the transducers’ frequency spectrum. According to the ultrasound pulse-echo test, the SFP and MFP transducers had the same center frequency of 11 MHz, and the −6 dB bandwidths of the SFP and MFP transducers were 91% and 109%. 

**Figure 12:**
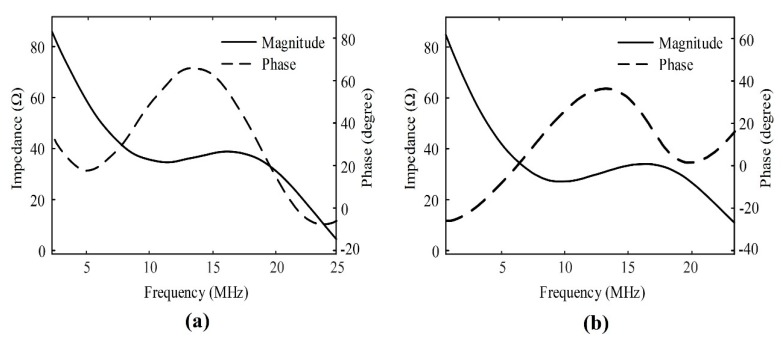
The measured electrical impedance magnitude and phase of (**a**) the single focal point transducer and (**b**) a seven-focal-point transducer.

Figure 12 shows the measured electrical impedance (magnitude and phase) of the fabricated SFP and MFP transducers. In the SFP transducer, the electrical impedance was measured at a magnitude of 36 Ω and a phase angle of 46° at 11 MHz. In the MFP transducer, the electrical impedance was measured at a magnitude of 32 Ω and a phase angle of 15° at 11 MHz. 

**Figure 13:**
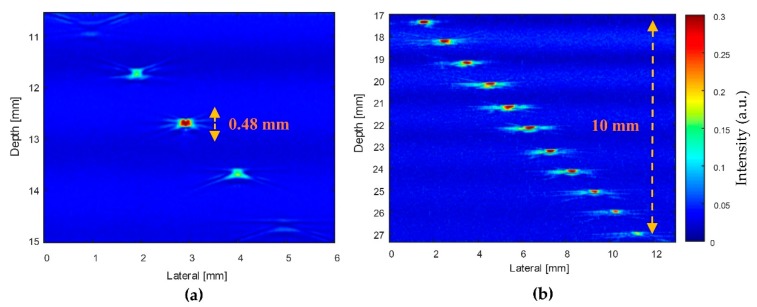
Ultrasound images of the wire phantoms obtained using (**a**) the SFP transducer and (**b**) the MFP transducer.

Figure 13 shows B-scan images of the wire phantom attained using the ring-shaped transducers. The wires were positioned in the transducers’ focal zones and produced the bright points in the image; otherwise, they appeared as blurred points in the images. In the ultrasound image of the 11 MHz SFP transducer (Figure 13a), only a single bright point at a depth of 12.7 mm was displayed, because the focal length of the SFP transducer was 12.7 mm. Using the seven-focal-point transducer, the ultrasound image of the wire phantom at 11 MHz was captured, as shown in Figure 13b. We observed that the MFP transducer displayed eleven bright points, showing a large focal zone (10 mm) for deeper images, which is significantly higher than the SFP transducer’s focal zone (0.48 mm). 

**Figure 14:**
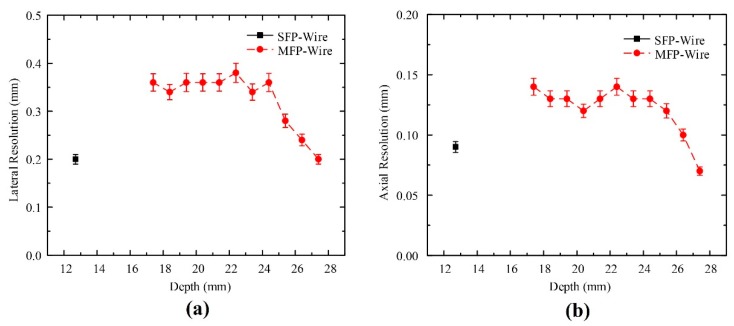
Ultrasound (**a**) lateral and (**b**) axial resolution measurements versus depth in the wire phantoms of the SFP (solid line) and MFP (dashed line) transducers. Error bars denote 95% confidence.

Figure 14 shows the lateral and axial resolutions of the SFP and MFP transducers. Using the wire phantom target, the spatial resolution of transducers was determined at full width at half maximum. At a depth of 12.7 mm, the SFP transducer had lateral and axial resolutions of 200 µm and 90 µm, respectively. In contrast, the MFP transducer can capture wire images at depths from 17.4 mm to 27.4 mm. The measured lateral resolutions at depths from 17.4 mm to 24.4 mm had a similar value of 360 µm. Lateral resolutions decreased linearly to 200 µm at a depth of 27.4 mm (Figure 14a). The measured axial resolution of the MFP transducer had a similar value of 140 µm at depths from 17.4 mm to 25.4 mm (Figure 14b). 

Although the MFP transducer has weak lateral and axial resolutions compared to SFP transducer, the MFP transducer can maintain good image quality at different depths due to its large focal zone. In case of the SFP transducer, the focal length and aperture diameter are 12.7 mm and 12 mm, from which a good F-number (focal length/aperture diameter: 12.7/12 = 1.05) could be acquired. Meanwhile, the MFP transducer has seven F-numbers from 4.4 mm to 2.3 mm, as shown in Table 2. Axial resolution is affected by the F-number, speed of sound, and center frequency of the transducer (Axial resolution = speed of sound x F-number/center frequency). A smaller F-number contributes to the improvement of the axial resolution. 

### 3.2. Penetration Depth in Photoacoustic Microscopy Images

**Figure 15:**
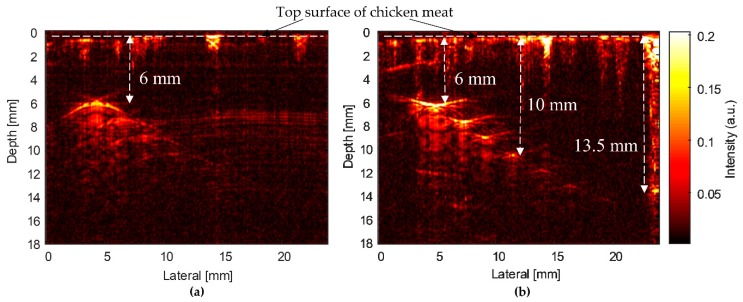
PAM images of the CN phantom acquired using (**a**) SFP and (**b**) MFP transducers. The penetration depths of the SFP and MFP transducers are 6 mm and 10 mm.

Figure 15 shows the PAM images of the CN phantom obtained using two transducer types. The SFP transducer’s PAM image displayed only one bright point at a depth of 6 mm (Figure 15a). In this case, a penetration depth of 6 mm was measured from the top surface of chicken meat to the image depth, in which the first needle was placed in the chicken tissue. The deeper needles’ images were not displayed clearly. The MFP transducer captured the PAM image of the first four needles at depths from 6 mm to 10 mm (Figure 15b). The brightest point is the first needle’s image at a depth of 6 mm. At a depth of 10 mm, the image of the fourth needle can be seen clearly. The image depth in chicken tissue was measured from the chicken meat’s surface to the farthest image point (0–10 mm). Therefore, the penetration depth of the MFP transducer in this CN phantom was measured to be 10 mm. However, blood vessels in the chicken phantom can absorb laser energy, which generates ultrasound waves and reflected signals to the transducer for forming images. Figure 15b shows the PAM image of the blood vessels at the top surface of the chicken meat, at the depth of 3 mm above the first needle, and at the right side from a depth of 0 to 13.5 mm. In this case, the penetration depth was measured to be 13.5 mm. Therefore, in this phantom, the MFP transducer’s penetration depth for the stainless steel needles is 10 mm and the penetration depth for chicken blood vessels is 13.5 mm. 

**Figure 16:**
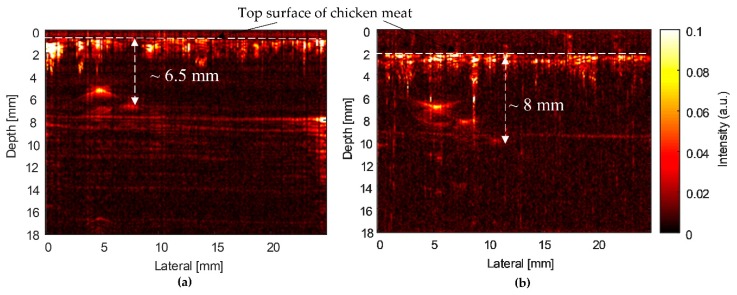
PAM images of the CHb phantom acquired using (**a**) SFP and (**b**) MFP transducers.

Figure 16 shows the PAM images of the CHb phantom obtained using two fabricated transducers. The SFP transducer’s PAM image displayed only a single bright point at a depth of 5 mm (Figure 16a). Therefore, the penetration depth of the SFP transducer in this phantom was measured to be 5 mm. The MFP transducer’s PAM image displayed three bright points at depths from 6.5 mm to 10 mm (Figure 16b). Therefore, the penetration depth of the MFP transducer in the CHb phantom is 8 mm, which was measured from the chicken meat’s surface to the third image point.

Table 4 summarizes the transducers’ parameters used in imaging systems. One type of piezoelectric material was designed for the same values of center frequency and different focal zones for two different transducers.

**Figure 17:**
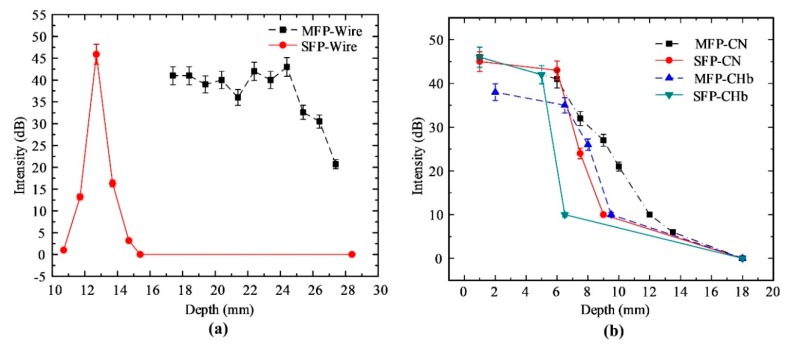
Target intensity versus depth for log-compressed (**a**) ultrasound images in the wire phantom and (**b**) photoacoustic images in the CN phantom and CHb phantom of the SFP (solid line) and MFP (dashed line) transducers. Error bars denote 95% confidence.

Figure 17 shows the mean intensity depth profiles for two transducers in the wire phantom, CN phantom, and CHb phantom. For the ultrasound image of the wire phantom shown in Figure 13, the intensity was considerably high at the focus depth of the transducers, and many more ultrasound waves reflected to the transducers (Figure 17a). The SFP transducer has only one focal point; therefore, the highest intensity was concentrated at a focal depth of 12.7 mm, whereas other depths had lower intensities. In case of the MFP transducer, a seven-focal-point transducer created a larger focal zone, which showed similar intensities at depths from 18.4 mm to 24.4 mm. For the PAM images of the CN and CHb phantoms shown in Figure 15 and Figure 16, the intensity decreased proportionally with depth in Figure 17b. However, the intensities of the PAM image from the MFP transducer were generally lower in the CHb phantom, because light absorbed by Hb is weaker than that absorbed by a needle in a chicken tissue. Owing to light diffusion in the chicken tissue, the intensity at a depth of 10 mm in the CHb phantom was 10 dB, whereas that in the wire phantom was 28 dB at the same depth. 

## 4. Discussion

The feasibility of extending the depth-of-field in PAM imaging using a custom MFP transducer was investigated in this study. The goal of this study was to harness the advantages of a single piezoelectric element for designing an MFP transducer for large-depth image applications. The SFP and MFP transducers were designed to compare the different features through their structures. The conventionally focused transducer created only one focal point with a short focal zone. To extend the length of the focal zone, the front face structure of the MFP transducer was designed with many spherically-focused surfaces, which produced many focal points and different focal zones. For the MFP transducer, the B-scan image was obtained by scanning a transducer along the X-axis over the wire phantom-based motion control system. The ultrasound data were collected from a single-channel ultrasonic pulser/receiver and plotted using the Matlab software without applying SAFT processing as with multi-element systems. 

For ultrasound and PA applications, a spherically focused transducer can enhance the sensitivity to detect small defects within objects or living tissues. At the same center frequency of 11 MHz, the MFP transducer had a focal zone of 10 mm and the SFP transducer had a focal zone of only 0.48 mm, which was demonstrated by ultrasound images of the wire phantom. 

For biomedical imaging applications, the PAM images of the CN phantoms in Figure 15 were obtained by two types of transducers. For the SFP transducer, the measured penetration depth and intensity were 6 mm (Figure 15a) and 43 dB (Figure 17b), respectively. Meanwhile, the measured penetration depth of the MFP transducer’s CN phantom image was 10 mm (Figure 15b) and the intensities were decreased from the phantom surface to the farthest image point (46–10 dB), as represented in Figure 17b. In the case of the Hb-filled tubes embedded in chicken tissue (Figure 16), the penetration depths of the SFP and MFP transducers were detected at 6.5 mm and 8 mm, respectively. The measured intensity at the focused depth of the CHb phantom image using the SFP transducer was 43 dB (Figure 17b), whereas in the MFP transducer, it decreased from the phantom surface to the farthest image point (35–10 dB), as represented in Figure 17b. In the same chicken tissue, needles absorbed much more laser light than Hb; therefore, at a deeper depth (10 mm) in the chicken tissue, the ultrasound waves still reflect to the MFP transducer to construct images. From the structure of the MFP transducer, the focal zone length can be extended by increasing the number of focal points and the aperture size.

Using phantoms (wire phantom, CN phantom, and CHb phantom), the performance of the MFP transducer was demonstrated to be suitable. However, B-scan images produced by a fixed focal point transducer can still benefit from SAFT, as the non-focal point scatterers were displayed as arcs due to the finite width of the transducer field away from the focus. As evident from Figure 13, Figure 15 and Figure 16, the developed MFP transducer is not free from this artifact. Instead of the arcs produced by the SFP transducer in the wire phantom image (Figure 13a), the MFP transducer produced horizontal spreading (Figure 13b), which has a much better appearance than the arcs, but is still an artifact of the same nature as the arcs. For the B-scan images of the chicken phantoms (Figure 15 and Figure 16), the MFP transducer produces arcs similar to those produced by the SFP transducer. The effects of horizontal spreading in water vs. arcs in the chicken phantoms are probably because of the difference in the sound speeds between water and the chicken phantom. Both the arcs and the horizontal spreading can be reduced using SAFT, which will be more difficult to implement for the MFP transducer. 

Table 4 shows the measured spatial resolution of transducers. However, the axial resolution of the MFP transducer can be improved at greater depths. This is because the axial resolution has an effect on the transducer’s parameters such as the center frequency and F-number. The MFP transducer has seven spherically-focused surfaces, which generate various focus depths with different F-numbers. The bigger aperture diameter has a smaller F-number (focal length/aperture diameter). The smaller F-number and the bigger center frequency results in a better axial resolution (Axial resolution = speed of sound x F-number/center frequency). The seventh part of the MFP transducer has the smallest F-number of 2.3 and the longest focal length of 28.87 mm. Therefore, the measured axial resolution of this part was 70 µm, which was the better axial resolution at deeper depths (Figure 17b).

## 5. Conclusions

This study described a novel design and evaluation of an MFP transducer with a significantly increased focal zone (10 mm) versus the SFP transducer (0.48 mm). The image of eleven phantom wires revealed the proposed MFP transducer’s extended focal zone. Additionally, the capacity to extend the focal zone for a larger-sized target was demonstrated, thereby enabling the imaging without the need for depth scans or any complex SAFTs. In ex vivo imaging, the penetration depth of CN and CHb phantoms increased to 10 mm and 8 mm, respectively. The intensity of the CN image decreased from the phantom surface to the farthest image point (4610 dB), whereas that in the CHb decreased from 35 to 10 dB. Specifically, the proposed seven-focal-point transducer is capable of generating seven focal zones along the axial direction simultaneously. Therefore, for large-depth imaging applications, MFP transducers have great potential. 

## Figures and Tables

**Table 1:** Properties of PVDF^1^ material.

Property	Value
Electromechanical coupling coefficient (*K_t_*)	0.15
Relative clamped dielectric constant $\varepsilon^{S}\;/\varepsilon$	11
Mechanical quality factor (*Q_m_*)	~20
Density (kg/m^3^)	1780
Longitudinal wave velocity (m/s)	2110
Acoustic impedance (MRayl)	3.9
Curie temperature (°C)	100
Melting temperature (°C)	160~180

^1^ Data reported by Piezo film sensor, AMP Inc, Valley Forge, PA.

**Table 2:** Parameters of the MFP transducer.

Part Number *i* (*i* = 1–7)	Focal Length *R_i_* (mm)	Aperture Diameter *D_i_* (mm)	*F*-Number (*R_i_/D_i_*)	Active Area (mm^2^)
1	20.00	4.60	4.3	1.256
2	21.49	6.49	3.3	1.256
3	22.97	7.94	2.8	1.256
4	24.45	9.16	2.6	1.256
5	25.92	10.24	2.5	1.256
6	27.40	11.21	2.4	1.256
7	28.87	12.10	2.3	1.256

**Table 3:** The distribution of the focal zones of the MFP transducer.

Focal Zone	Range of Focus (mm)	Length of Focus (mm)	Overlap Length (mm)
F_Z1_	18.50 – 22.20	3.70	–
F_Z2_	20.18 – 22.80	2.61	2.02
F_Z3_	21.97 – 23.98	2.01	0.83
F_Z4_	23.59 – 25.31	1.72	0.39
F_Z5_	25.15 – 26.71	1.55	0.16
F_Z6_	26.67 – 28.19	1.45	0.04
F_Z7_	28.18 – 29.50	1.38	0.01

**Table 4:** Operating parameters of ultrasonic transducers.

Parameter	SFP Transducer	MFP Transducer
Center frequency (MHz)	11	11
-6 dB Bandwidth (%)	91	109
SNR (dB)	38.7	34
Focal length (mm)	12.7	18.5 - 29.5
Axial resolution (µm)	90	140 - 70
Lateral resolution (µm)	200	360 - 200
Focal zone (mm)	0.48	10
